# Large-scale modulation of reconstituted Min protein patterns and gradients by defined mutations in MinE’s membrane targeting sequence

**DOI:** 10.1371/journal.pone.0179582

**Published:** 2017-06-16

**Authors:** Simon Kretschmer, Katja Zieske, Petra Schwille

**Affiliations:** 1 Department of Cellular and Molecular Biophysics, Max Planck Institute of Biochemistry, Martinsried, Germany; 2 Graduate School of Quantitative Biosciences, Ludwig-Maximilians-Universität, München, Germany; Oregon State University, UNITED STATES

## Abstract

The *E*. *coli* MinDE oscillator is a paradigm for protein self-organization and gradient formation. Previously, we reconstituted Min protein wave patterns on flat membranes as well as gradient-forming pole-to-pole oscillations in cell-shaped PDMS microcompartments. These oscillations appeared to require direct membrane interaction of the ATPase activating protein MinE. However, it remained unclear how exactly Min protein dynamics are regulated by MinE membrane binding. Here, we dissect the role of MinE’s membrane targeting sequence (MTS) by reconstituting various MinE mutants in 2D and 3D geometries. We demonstrate that the MTS defines the lower limit of the concentration-dependent wavelength of Min protein patterns while restraining MinE’s ability to stimulate MinD’s ATPase activity. Strikingly, a markedly reduced length scale—obtainable even by single mutations—is associated with a rich variety of multistable dynamic modes in cell-shaped compartments. This dramatic remodeling in response to biochemical changes reveals a remarkable trade-off between robustness and versatility of the Min oscillator.

## Introduction

Living systems establish spatiotemporal patterns on scales ranging from molecules to populations [1, 2]. These patterns orchestrate basic life processes including cell polarization, cytokinesis and animal development [3–6]. Pioneering theoretical studies have shown that complex patterns can emerge in reaction-diffusion systems with as little as two interacting components under certain functional conditions [7, 8]. Experimentally, an elegant way to study biological pattern formation is based on reconstituted minimal systems, which allow control over important system parameters [9, 10]. Here, we use the reconstituted Min oscillator to address how large-scale protein patterns and gradients are modulated by mutations in the underlying self-organizing proteins.

The *E*. *coli* Min oscillator is an archetypical model system for protein self-organization. At its mechanistic core, it is based on the ATP-dependent switching of the proteins MinD and MinE between an aqueous phase and a lipid membrane [11]. In this context, the membrane serves as a template for reversible protein association and plays a quasi-catalytic role in pattern formation, analogous to heterogeneous catalysis reactions [5, 12]. On a molecular level, MinD interacts cooperatively with the membrane upon ATP-dependent dimerization [13, 14]. Conversely, MinE antagonizes MinD’s accumulation on the membrane via stimulation of MinD’s ATPase activity, resulting in dissociation of MinD monomers from the membrane [13, 15]. This cycle of autocatalytic membrane binding of MinD and subsequent release by MinE constitutes the key mechanism of Min protein dynamics.

In *E*. *coli*, the Min system spatially regulates cytokinesis by localizing the division machinery to the cell middle through an inhibitory gradient of MinC formed by pole-to-pole oscillations of MinD and MinE [16–19]. *In vitro*, Min proteins self-organize into traveling waves with a defined wavelength and velocity on flat lipid bilayers, whose dimensions are orders of magnitude larger than individual waves [11]. Recently, we reconstituted gradient-forming oscillations in cell-shaped microcompartments, which mimic the rod-like shape of *E*. *coli* but are scaled to the roughly ten times larger spatial dimensions of Min patterns *in vitro* [20, 21]. It has been characterized extensively how the geometry of the surrounding boundaries modulates Min protein dynamics [21–26]. Likewise, several studies investigated physical and biochemical influences on reconstituted Min protein patterns [27–30]. However, an in-depth understanding of how molecular motifs determine the properties of Min protein patterns and gradients, which are several orders of magnitude larger than the proteins, has so far remained elusive.

Due to the membrane’s central role in pattern formation, the protein domains responsible for lipid interaction are particularly interesting motifs of Min proteins. Both MinD and MinE interact with the membrane via amphipathic helices at their C- and N-termini, respectively [31, 32]. The role of cooperative MinD membrane binding in pattern formation is intuitive, allowing MinD to accumulate in a self-enhanced fashion on the membrane. However, the core antagonistic function of MinE would in principle be possible without direct membrane interaction, but by solely binding and subsequently detaching MinD via stimulation of its ATPase activity.

Even though MinE’s MTS has been investigated previously, its precise role in pattern and gradient formation has remained ambiguous. While it was suggested theoretically that MinE membrane interaction is important for robust pattern formation [23, 33], many mathematical models display regular Min protein dynamics even in its absence [34–36]. On the other hand, *in vivo* experiments showed that mutations in MinE’s MTS are associated with severe cell division defects [31]. Furthermore, previous *in vitro* data suggested that mutations lead to either disordered patterns or traveling waves with altered characteristics on flat membranes, dependent on the mutation [27, 29]. Additionally, we recently observed that pole-to-pole oscillations in cell-shaped microcompartments are compromised upon a deletion in MinE’s MTS [21]. While these experimental studies indicate an important role of MinE’s MTS, its precise role is still unclear due to the discrepancy of theoretical predictions and the range of apparently contradictory effects described *in vitro*. Thus, important questions remain: Is direct MinE membrane interaction indispensable for pattern and particularly gradient formation or does it serve a modulatory role? If regular pole-to-pole oscillations are compromised, which other modes may emerge in cell-like geometry and how do they affect functional gradient formation? Finally, how stable is the Min-based positioning system against biochemical variations, i.e. can relatively simple biochemical changes like single mutations result in a large-scale remodeling of the Min oscillator, and if so, how?

Previous conclusions on the role of MinE’s MTS in pattern and gradient formation were based on *in vitro* experiments with only one mutant at a particular concentration in a given geometric setup that was different in each case [21, 27, 29]. However, emergent behaviors in reaction-diffusion systems are generally sensitive to changes in parameter values and typically depend on the interplay of various factors. Thus, the different results regarding direct MinE membrane interaction are hard to compare and general conclusions difficult to draw. Therefore, we report here the first *in vitro* study focusing on MinE’s direct membrane interaction that systematically and comprehensively explores variations in protein sequence, concentration and assay geometry, with regard to pattern and in particular functional gradient formation. With this approach, we uncover that direct MinE membrane interaction fundamentally regulates the lower limit of the concentration-dependent length scale of Min protein patterns while restraining MinE’s capacity to stimulate MinD’s ATPase activity. These effects are associated with a rich variety of unusual modes in cell-like geometry, characterized by severe defects in gradient formation. Strikingly, even single mutations in MinE’s MTS cause a large-scale remodeling of the Min oscillator, revealing a trade-off between versatile dynamic behavior and robustness against biochemical changes.

## Results

### Direct MinE membrane interaction shifts the lower limit of the concentration-dependent length scale of Min protein patterns

Membrane interaction of MinE has been suggested to be mediated both by conserved hydrophobic residues (L3, L4, F6, F7, L8), which are inserted into the core of the lipid bilayer, as well as a cluster of cationic residues (R10, K11, K12), apparently interacting electrostatically with anionic lipid head groups [31, 37, 38]. While an initial reconstitution study of a mutant with impaired electrostatic interactions (MinE R10G/K11E/K12E) appeared unable to self-organize into planar surface waves [27], a mutant that lacked hydrophobic residues but left some of the cationic residues intact (MinE^11-88^) formed surface waves [29]. This discrepancy still left doubts whether or not direct MinE membrane interaction was required for wave formation. Therefore, we engineered a MinE mutant lacking the entire MTS (MinE Δ(2–12)) and thus, being impaired in both hydrophobic and electrostatic interactions.

**Fig 1:**
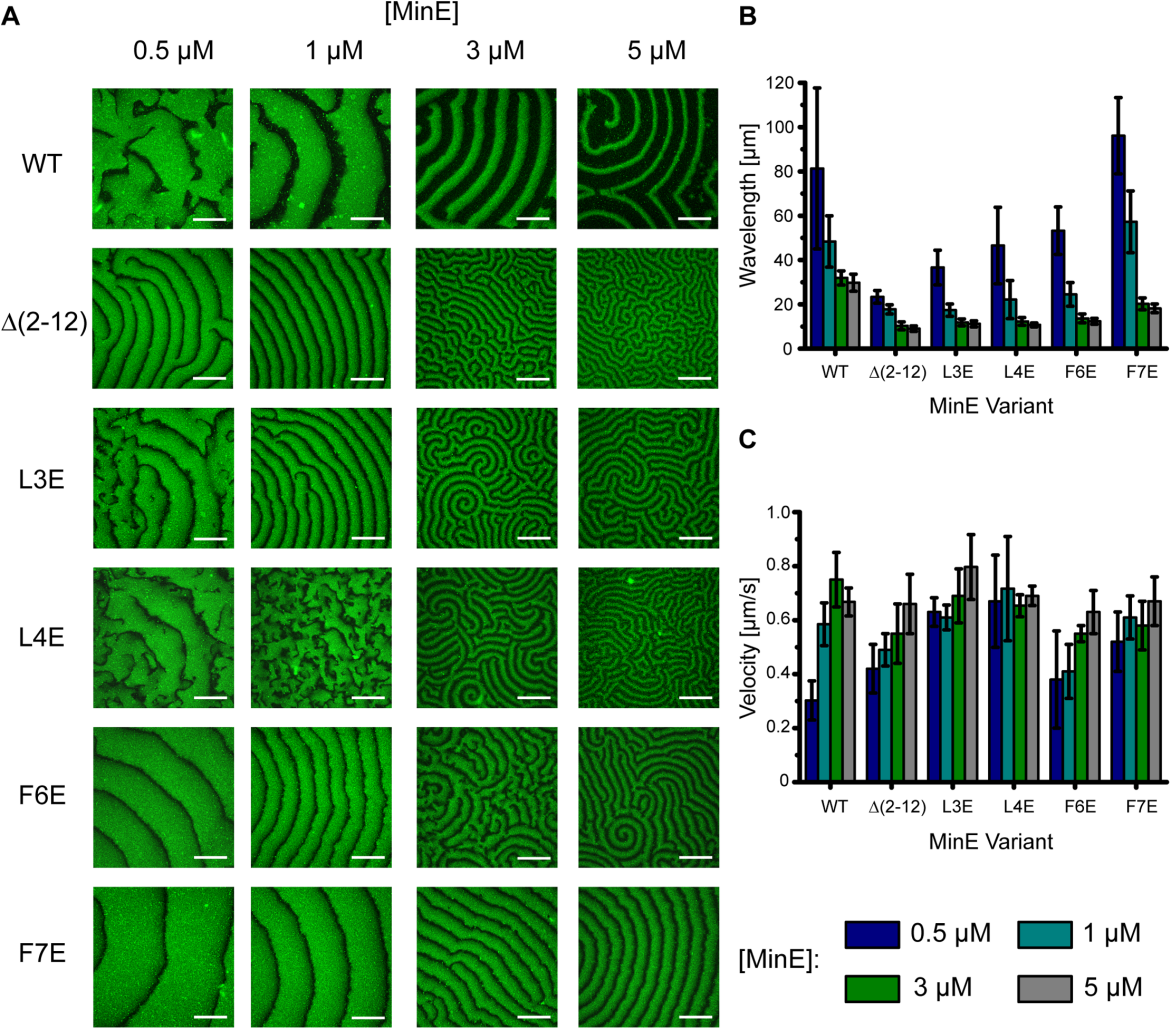
Truncation or mutation of MinE’s membrane targeting sequence decreases the lower limit of the length scale of Min protein patterns. **(A)** Confocal images of self-organized WT and Δ(2–12), L3E, L4E, F6E, F7E mutant waves on flat membranes. WT MinE and mutant proteins were titrated from 0.5 to 5 μM (MinD at 1 μM with 20% eGFP-MinD). Scale Bar: 50 μm. Dependence of the mean **(B)** wavelength and **(C)** velocity of WT and mutant waves on MinE concentration (MinD at 1 μM). Error bars represent standard deviation (N ≥ 3) from at least three independent experiments.

Strikingly, MinE Δ(2–12) still supported self-organization into regular spiral and traveling waves, when reconstituted with MinD and ATP on a supported lipid bilayer (SLB) (Fig 1A and S1 Movie). This demonstrates that direct MinE membrane interaction is dispensable for pattern formation *per se*, consistent with mathematical models that do not require the incorporation of direct MinE membrane interaction [35, 39].

Remarkably, we observed a reduced length scale of wave patterns for MinE Δ(2–12) compared to WT MinE (Fig 1A and 1B). A similar effect has been reported for MinE^11-88^ in an SLB-coated flow cell [29]. However, the severity of the reported change was still unclear, as the wavelength of Min patterns is known to also depend on the [MinE]/[MinD] ratio [11]. Therefore, we systematically varied the [MinE]/[MinD] ratio and measured the wavelength of the truncation mutant. With this approach, we determined that the wavelength of MinE Δ(2–12) could be reduced to as low as roughly 10 μm, compared to around 30 μm for the WT (Fig 1A and 1B). On the other hand, the velocity of the mutant waves saturated on a similar level compared to WT MinE. Vecchiarelli et al. reported an increased wave velocity for MinE^11-88^ compared to WT MinE at one tested concentration in their flow-cell setup [29]. These results are not directly comparable due to potential effects of flow as well as the unknown local concentrations within the flow cell that give rise to the patterns. However, despite these differences, the reported mutant velocity is in the same range as the maximum velocity for all of our mutant proteins, suggesting a general agreement. In summary, impairing MinE membrane interaction via truncation strongly reduced the lower limit of the concentration-dependent wavelength, allowing Min protein waves to assume small length scales impossible to obtain with the WT even at increased MinE concentrations (Fig 1A–1C).

To further investigate the effects of reduced membrane affinity on Min patterns, we focused on hydrophobic membrane interactions of specific amino acids in the MTS. For this, we analyzed Min protein patterns of MinE mutants with single hydrophobic residue mutations. Specifically, residues L3, L4, F6 or F7 were substituted by glutamate to disrupt the amphipathicity of the MTS. *In vivo* experiments showed that these mutants are impaired in membrane interaction [31], which we corroborated *in vitro* using a liposome co-sedimentation assay (S1 Fig). Strikingly, although the patterns varied slightly between the different mutants at a given concentration, we observed that the characteristic reduction in the lower limit of the wavelength was observed for all mutants, similar to the truncation mutant (Fig 1A and 1B). Taken together, our results demonstrate that MinE’s MTS defines the lower limit of the concentration-dependent wavelength, and that mutations of even single hydrophobic residues can dramatically reduce the length scale of Min protein patterns.

### Direct MinE membrane interaction restrains MinE’s capacity to stimulate MinD’s ATPase activity

The length scale of Min protein patterns represents an emergent property of a self-organizing system. As changes in such observables often depend directly or indirectly on different parameters, we investigated how mutations in the MTS affect MinE’s core function of stimulating MinD’s ATPase activity, which is directly responsible for triggering MinD detachment from the membrane [13, 15]. For this, we performed MinD ATPase stimulation assays in the presence of liposomes with the truncation mutant and all four mutants with individual substitutions of hydrophobic residues.

**Fig 2:**
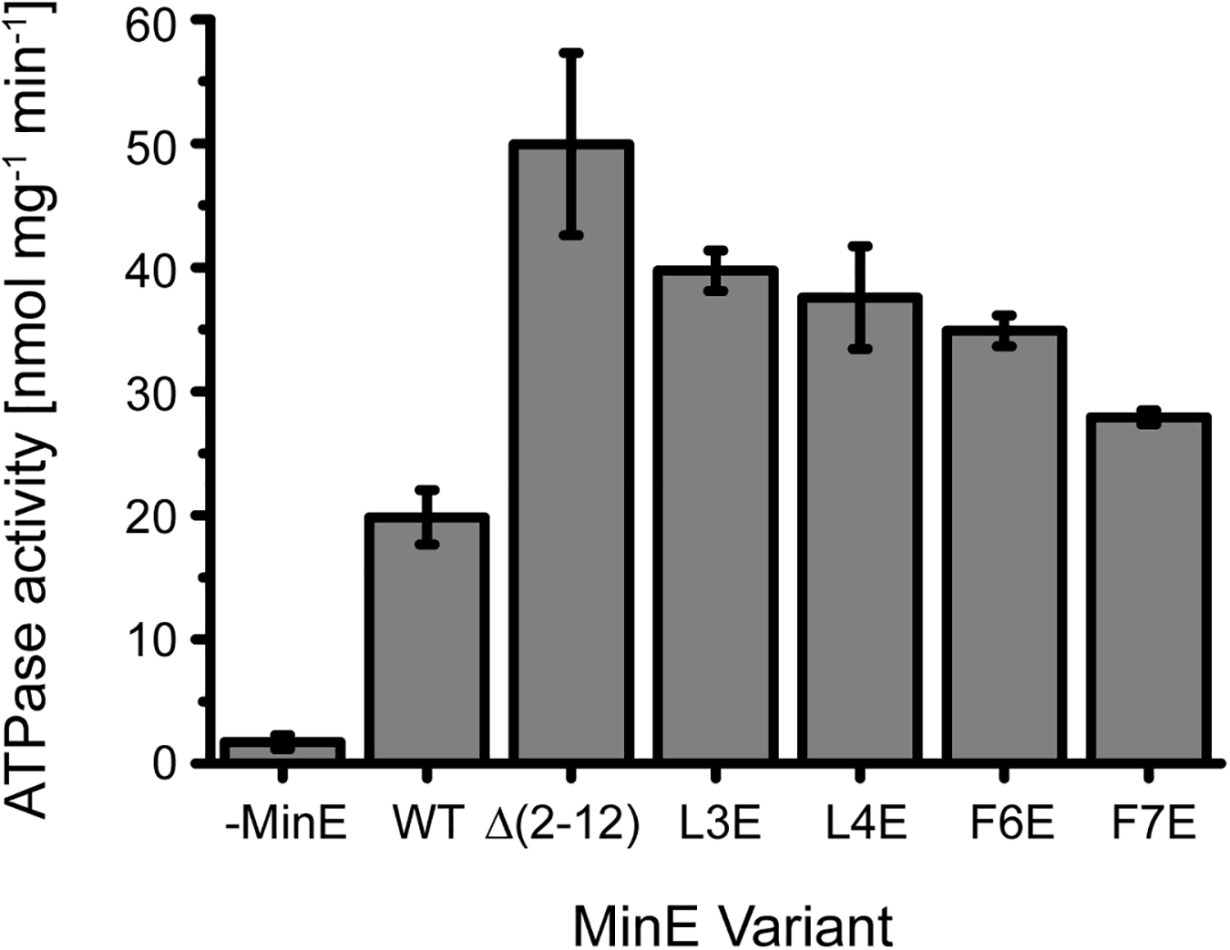
Mutation or truncation of MinE’s membrane targeting sequence causes higher bulk stimulation of MinD’s ATPase activity, indicating increased antagonistic potential of MinE in the absence of its direct membrane interaction. ATPase stimulation assay with WT MinE or MinE ∆(2–12), L3E, L4E, F6E, F7E using 4 μM MinD, 4 μM MinE and 0.2 mg/mL small unilamellar vesicles made of *E*. *coli* polar lipids. Error bars represent standard deviation from three independent experiments.

Strikingly, we observed that all five mutants stimulated MinD’s ATPase activity to a significantly higher level than the WT (Fig 2). This previously unknown increase in ATPase stimulation by single mutations in the MTS’s hydrophobic residues is consistent with the increased MinD ATPase stimulation reported for MinE R10G/K11E/K12E, which is impaired in electrostatic membrane interaction [37]. On the other hand, Vecchiarelli et al. observed only a slight, non-significant difference in the ATPase stimulation for MinE^11-88^ [29]. However, these experiments were performed at different concentrations and temperature as well as in a different assay from ours, impeding direct comparability. Mechanistically, the effectively higher ATPase rate could be due to faster MinE detachment following stimulation of one MinD dimer’s ATPase activity and thus shorter delay before binding the next one.

In conclusion, direct membrane interaction appears to restrain MinE’s capacity to stimulate MinD’s ATPase activity and thereby also to antagonize MinD accumulation on the membrane. Conversely, mutations in MinE’s MTS alleviate this restrain. Thus, the increased ATPase stimulation observed with the mutants indicates a more efficient detachment of MinD from the membrane, which may explain the shorter wavelength of the mutant patterns.

### Direct MinE membrane interaction shapes the Min gradient by adapting Min protein dynamics to cell-like geometry

As impaired MinE membrane interaction reduced the length scale of Min waves while still supporting pattern formation, the role of MinE membrane binding in Min oscillation and gradient formation was an outstanding question. Our previous study with a mutant lacking the MTS’s hydrophobic patch indicated that regular pole-to-pole oscillations in cell-like geometry are compromised without direct MinE membrane binding [21]. However, it has remained unclear which specific dynamic modes can emerge in the absence of MinE membrane interaction and in particular, how each of them affects gradient formation. Furthermore, it was unclear how sensitive the Min oscillator is to single mutations in the membrane targeting sequence. Therefore, we reconstituted MinE L3E together with MinD and ATP under physiological conditions in cell-shaped compartments.

**Fig 3:**
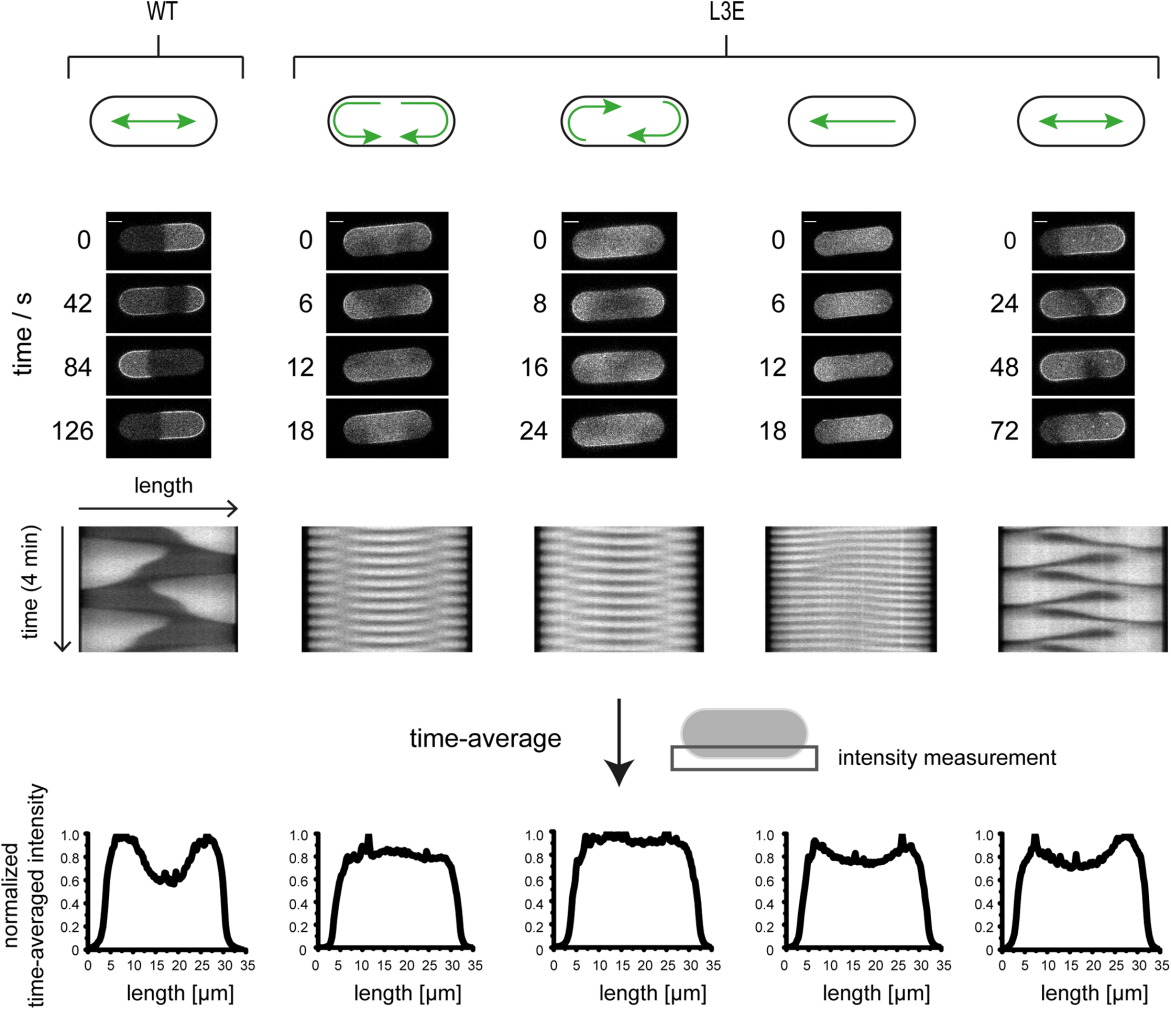
Mutation of MinE’s membrane targeting sequence leads to unusual dynamics and defects in gradient formation in cell-shaped compartments. WT and L3E panels show representative time-lapse images, kymographs along the compartment length as well as the time-averaged fluorescence intensity, which was measured along a compartment edge. The L3E mutant exhibited diverse dynamical modes observed in different compartments under the same experimental conditions. These mutant dynamics comprised (from left to right) bi- or unidirectional rotations, traveling waves and irregular pole-to-pole oscillations. All images at 1 μM MinD with 20% eGFP-MinD and 1 μM MinE. Scale Bar: 5 μm. The compartments were 35 μm long, 10 μm wide and 10 μm deep.

Remarkably, in contrast to the typical pole-to-pole oscillations for WT MinE or the irregular dynamics reported previously [21], the L3E mutant supported a rich diversity of dynamic modes (Fig 3, S2–S6 Movies). Notably, these modes emerged in different compartments under the same experimental conditions and occasionally even alternated within the same compartment. This diversity of *in vitro* Min protein dynamics supports the notion of multistability, previously reported *in vivo* and *in silico* [34, 40].

We observed four major types of defined dynamics for the L3E mutant. Besides pole-to-pole oscillations, which appeared irregular compared to WT oscillations, traveling waves as well as striking rotational modes emerged (Fig 3, S3–S6 Movies). In the rotational dynamics, MinD either split into two concurrent and bidirectional rotations via both poles or performed unidirectional rotations around the entire compartment periphery (S3 and S4 Movies). Interestingly, bidirectional rotations appeared like a short-axis oscillation in kymographs along the compartment width (S2 Fig). Importantly, WT oscillations produced a clear gradient with a depth of up to 50%. In contrast, the rotational modes observed with MinE L3E did not display a time-averaged gradient. Moreover, the traveling waves and oscillations displayed by MinE L3E formed weaker gradients with a depth of up to only around 70% (Fig 3). Thus, the different dynamic modes observed for MinE L3E either completely abolished or substantially compromised gradient formation. This indicates that MinE’s MTS mediates functional gradient formation by selecting regular pole-to-pole oscillations and suppressing alternative dynamics under physiological conditions in cell-like geometry.

To confirm that unusual dynamics also emerge for other mutations in the MTS, we reconstituted MinE Δ(2–12) and F6E mutant dynamics in cell-shaped microcompartments (S3 and S4 Figs). We observed similarly unusual dynamics with the most notable difference being the observed fraction of the respective modes (S3–S5 Figs).

Taken together, our results demonstrate that MinE’s capacity to directly interact with the membrane plays a key role in selecting the modes of Min protein dynamics and thereby adapting the Min oscillator for gradient formation in cell-like geometry. Intriguingly, a rich diversity of dynamic modes can emerge even without direct MinE membrane interaction. Finally, even a single mutation in MinE’s MTS causes a large-scale remodeling of the Min oscillator.

## Discussion

**Fig 4:**
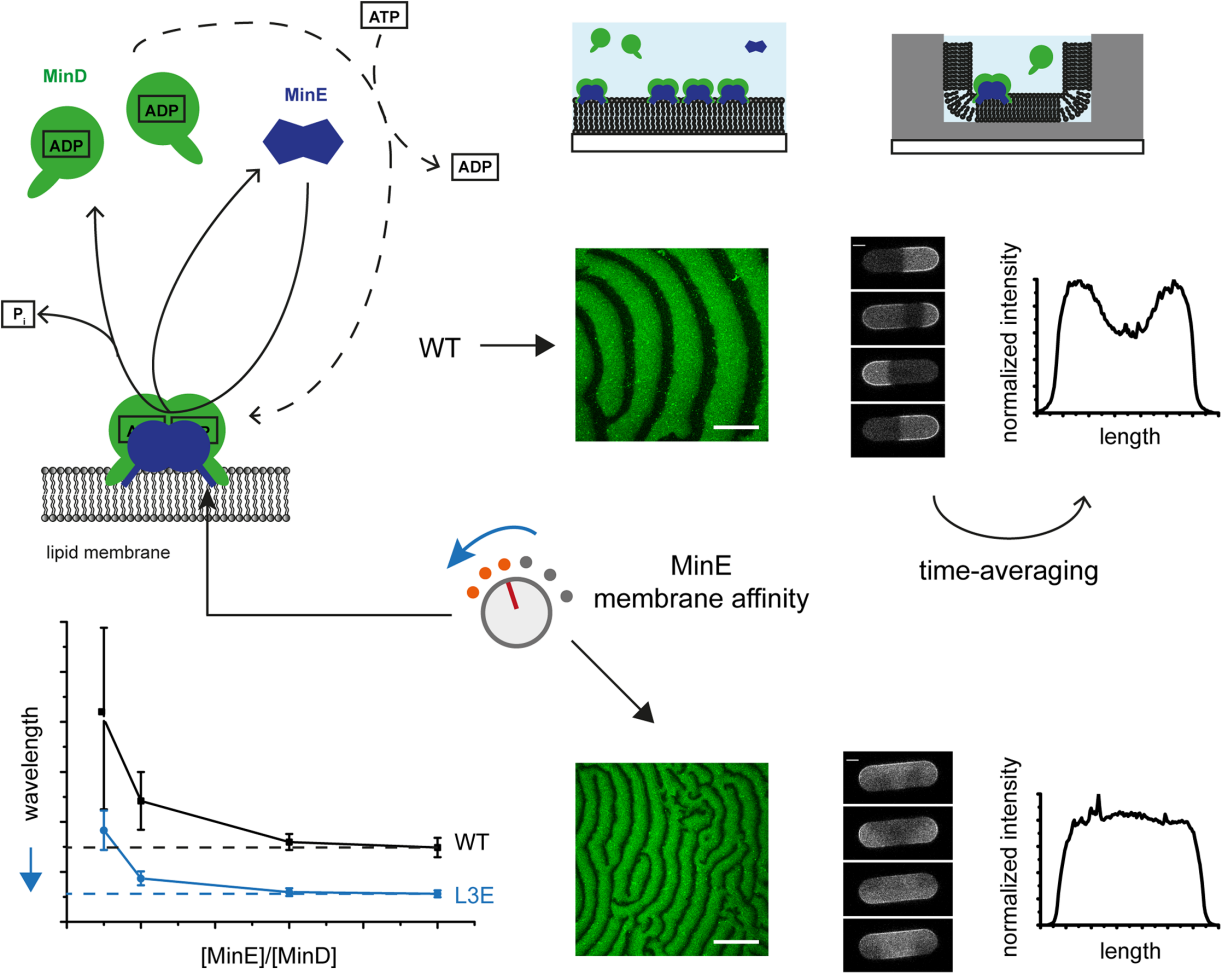
Large-scale modulation of the Min oscillator by reducing MinE’s membrane affinity. Biochemical alterations, such as single mutations, in MinE’s membrane targeting sequence cause a marked reduction in the lower limit of the length scale of Min protein patterns along with unusual dynamic modes in cell-like geometry. Thus, the Min oscillator is both highly versatile and sensitive to biochemical changes. Scale Bars: 50 μm (left) and 5 μm (right). P_i_: inorganic phosphate. Units and values on graphs are left out for simplicity (see Figs 1 and 3 for data).

With this study, we advance our previous research using the Min oscillator as a model system for protein self-organization and gradient formation [11, 21]. Specifically, we dissected how large-scale protein dynamics are modulated by changes in the membrane targeting sequence of a pattern-forming protein. We focused on the role of direct MinE membrane interaction, which has been the subject of considerable debate, due to apparently conflicting experimental as well as theoretical data [27, 29, 33, 35]. By systematically varying protein sequence, concentration and assay geometry, we provide the first systematic study to directly relate reduced length scales of mutant protein patterns to different dynamic modes in cell-like geometry (Fig 4).

The original notion that direct MinE membrane interaction is required for regular wave formation was based on the observation of irregular patterns emerging with MinE R10G/K11E/K12E at a single [MinE]/[MinD] concentration ratio [27]. However, we have shown here that both WT MinE and different mutant proteins form regular surface waves at certain concentrations, while patterns appear more irregular at others. This highlights that it is important to test for pattern formation in a wide range of [MinE]/[MinD] ratios and that–besides mutations—MinD and MinE protein concentrations are key factors in determining self-organization. Our observation of surface waves with all mutants, and especially the truncation mutant lacking the entire MTS, unambiguously demonstrates that direct MinE membrane interaction is not required for the formation of Min protein waves, in line with results reported for MinE^11-88^ [29].

Comparing our results from 2D and 3D reaction geometries showed that a reduced length scale of mutant protein patterns was accompanied by a diversity of unusual dynamic modes in cell-like geometry (Fig 4), namely rotations, traveling waves and irregular pole-to-pole oscillations. It is plausible to assume that the reduced wavelength and the unusual dynamics are due to a common underlying phenomenon, such as the mutants’ increased capacity to stimulate MinD’s ATPase activity (Fig 2). Moreover, it is probable that a large-scale adaptation between the length scale of Min protein patterns and the dimensions of the surrounding boundaries governs the selection of dynamic modes. Notably, it was shown that unusual dynamic modes emerge when Min proteins are subjected to non-natural geometries [21, 22, 24, 33]. In particular, rotations and traveling waves were recently observed when WT Min proteins were incubated in chambers larger than the ones favoring pole-to-pole oscillations [22]. Intriguingly, our approach uncovers similar dynamics through biochemical changes to the proteins themselves, while keeping the geometry fixed. These complementary results strongly indicate that a large-scale adaptation between the length scale of Min protein patterns and the dimensions of the surrounding boundaries governs the selection of dynamic modes. Accordingly, by defining the lower limit of the wavelength of Min protein patterns, MinE’s MTS would adjust the length scale of the protein patterns for robust function in cell-like geometry.

Previously, the observation of an altered wave profile for MinE^11-88^ on a flat membrane suggested that MinE’s MTS mainly functions as a means to allow “lingering” of MinE on the membrane upon detaching MinD [29]. Substantial lingering would prevent reattachment of MinD at the same pole and thereby enforce pole-to-pole oscillations. However, the geometry-dependent observation of unusual dynamics with WT MinE, capable of lingering, argues against its loss being the primary cause of unusual dynamics [22]. Additionally, we still observed a fraction of pole-to-pole oscillations even without direct MinE membrane interaction. However, these oscillations appeared somewhat irregular and the resulting gradient less defined compared to the WT, consistent with the altered waves on flat membranes, published in *vivo* data as well as the inability of Min proteins to form burst patterns in a flow cell in the absence of MinE membrane interaction [29, 37]. Thus, while a large-scale adaptation between the length scale of Min patterns and the surrounding geometry could select pole-to-pole oscillations as the primary mode, additional small-scale effects such as MinE lingering may fine-tune the oscillations to optimize gradient depth.

The notion that MinE’s MTS is not required for pattern formation but adapts Min protein dynamics to cell geometry also reconciles previously conflicting theoretical and experimental observations. Min oscillations can theoretically emerge even without direct MinE membrane interaction [35]. However, mutations in MinE’s MTS can still indirectly impair gradient formation through a mismatch between the length scale of protein patterns and cellular geometry. Thus, the geometry-dependent occurrence of pole-to-pole oscillations in mathematical models appears not at all inconsistent with abnormal dynamics and severe cell division defects observed *in vivo* for mutations in MinE’s MTS [31, 37].

Our systematic characterization of MinE mutants can also inform new mathematical models of pattern formation. While reaction-diffusion systems are known to respond strongly to changes in parameter values, it is vital to identify experimentally accessible properties as modulatory parameters and dissect how exactly they change the system’s behavior. Thus, our systematic analysis of the mutants’ wavelength and velocity, including their dependence on MinE concentration, can serve as a test bed for models aiming at a quantitative agreement between experiments and theory.

Finally, our results demonstrate that a large-scale remodeling of the Min oscillator can arise from relatively simple biochemical changes, such as single mutations in the membrane targeting sequence of a pattern-forming protein. On the one hand, this highlights that the Min system is relatively sensitive toward biochemical alterations and that gradient formation is easily impaired. On the other hand, the capability to change its dynamic behavior by single mutations makes the Min oscillator highly versatile, potentially allowing it to easily adapt to different geometries in the course of evolution. For example, whereas pole-to-pole oscillations would be impaired in *E*. *coli* cell geometry, a reduced length scale of protein patterns could enable the Min system to function in smaller-than-usual geometries. Future experiments could investigate such adaptability by systematically varying both the protein sequence and the surrounding geometry. Another important question is how the Min oscillator responds to alterations in other biochemical features of MinD and MinE. Emergent properties of self-organizing systems are typically determined by the interplay of various factors. Thus, it is likely that new dynamic behaviors will be uncovered and that similar effects to the ones here can be obtained through different kinds of perturbations. Interestingly, a trade-off between versatile dynamic behavior and sensitivity to biochemical changes has recently been reported for a reconstituted Ras signaling network [41], suggesting that this phenomenon might be widespread. The exploration of such trade-offs in biochemical networks as well as their careful balancing in the context of synthetic cell designs are intriguing future prospects.

## Materials and methods

### Plasmids

Plasmids for expression of MinE mutant proteins were generated with our previously described His-MinE expression vector [11]. All mutants with amino acid substitutions were made by site-directed mutagenesis using the GeneArt^®^ Site–Directed Mutagenesis System (Invitrogen, Carlsbad, CA, USA). The MinE truncation mutant lacking amino acids 2–12 was generated with the GeneArt^®^ Seamless Cloning and Assembly Enzyme Mix (Invitrogen, Carlsbad, CA, USA). All plasmids were sequenced to verify the mutations. Mutagenic primers used in this study are shown in S1 Table.

### PDMS microcompartments

The two-dimensional geometry of microstructures was designed by using the software AutoCAD. A chrome mask (Compugraphics Jena GmbH, Jena, Germany) was used to generate photoresist microstructures (photoresist: ma-P 1275, micro resist technology GmbH, Berlin, Germany) on top of Si wafers (Si-Mat, Kaufering, Germany) by photolithography. The wafer with the resist structures was coated with chlorotrimethylsilane (Sigma–Aldrich, St. Louis, MO, USA).

PDMS (Sylgard184, Dow Corning, Midland, MI, USA) base was mixed with cross-linker at a ratio of 9:1. The PDMS mixture was degased in a vacuum chamber and poured on top of the wafer. Glass cover slips were pressed into the liquid PDMS mixture on top of the Si-waver. The PDMS was cured on top of the wafer for three hours at 80°C and the glass coverslip with a layer of microstructured PDMS was carefully peeled of the Si-wafer, as described previously [21].

### Protein purification

MinD, eGFP-MinD as well as WT MinE and MinE mutants were expressed and purified as His-tagged fusions as described previously [11, 21]. After purification, any precipitates were removed by centrifugation in an MLA-130 rotor (Beckman Coulter, Brea, CA, USA) at 50,000 rpm for 30 min at 4°C. Protein quantification was performed by Bradford assay (Bio-Rad Protein Assay, Bio-Rad Laboratories Inc., Hercules, CA, USA). All protein samples were frozen in storage buffer (50 mM Hepes pH 7.25, 150 mM KCl, 10% glycerol, 0.1 mM EDTA, 0.2 mM TCEP; and for MinD 0.2 mM ADP) and stored at -80°C.

### Preparation of small unilamellar vesicles (SUVs)

*E*. *coli* polar lipids (Avanti Polar Lipids, Alabaster, AL, USA) in chloroform were dried under constant flow of nitrogen. After applying vacuum for 30 min, lipids were resuspended in SLB buffer (25 mM Tris-HCl pH 7.5, 150 mM KCl) to a final concentration of 4 mg/mL and incubated at 37°C for 1 h while vortexing every 20 min. Liposomes were then sonicated until clear and stored at– 20°C. Thawed SUVs were roughly 70 nm in average diameter, as determined by dynamic light scattering.

### Preparation of supported lipid bilayers (SLBs)

Supported lipid bilayers were generated by vesicle fusion of *E*. *coli* polar lipid SUVs as described previously [42]. For this, a suspension of 0.5 mg/mL SUVs in SLB buffer along with 3 mM CaCl_2_ was added to a plastic chamber with glass, or PDMS microcompartments at the bottom. Samples were incubated at 20°C and non-fused vesicles removed by washing the bilayers with SLB buffer. Before self-organization assays, SLB buffer was exchanged to Min buffer (25 mM Tris-HCl pH 7.5, 150 mM KCl, 5 mM MgCl_2_).

### Self-organization assay on flat supported membranes

Self-organization assays on flat supported lipid bilayers were performed essentially as described previously [11]. Briefly, purified MinD and MinE were added along with 2.5 mM ATP (F. Hoffmann-La Roche AG, Basel, Switzerland) in 200 μL Min buffer to SLBs prepared on glass.

### Self-organization assay in PDMS microcompartments

Self-organization assays in PDMS compartments were performed as described previously [20, 21]. After addition of proteins and ATP to SLBs prepared on PDMS, the sample was incubated until Min patterns could be observed. The volume of the buffer solution was then carefully reduced to trap assay components inside the compartments. The protein concentrations stated in the text refer to the concentrations before buffer aspiration. Note that, when the lower planes of compartments were imaged, the dimensions in the images appear smaller than in the top plane.

### Microscopy and image processing

Fluorescence imaging was performed with a ZEISS LSM780 confocal laser scanning microscope using a Zeiss C-Apochromat 40x/1.20 water-immersion objective (Carl Zeiss AG, Oberkochen, Germany). All images were processed using Fiji. Any brightness or contrast adjustments were uniformly applied to the entire image field or stack.

### Liposome co-sedimentation assay

The co-sedimentation assay was adapted from a previously published protocol [43]. 5 μM MinE mutants were incubated with 0.5 mg/mL small unilamellar vesicles in 50 μL Min buffer for 15 min at room temperature. Vesicles were then pelleted by centrifugation at 25000 rpm in a TLA-100 rotor (Beckman Coulter, Brea, CA, USA) for 10 min at room temperature. The supernatants were collected and the pellets resuspended in the original volume. Supernatant and pellet fractions were analyzed by SDS-PAGE, gels stained with InstantBlue Protein Stain (Expedion, Meridian, UK) and protein amounts estimated using Fiji.

### ATPase stimulation assay

Measurement of MinD’s basal and MinE-stimulated ATPase activity was performed with an ATP/NADH-coupled assay [14]. In this system, ATP hydrolysis is coupled to the conversion of phosphoenolpyruvate (PEP) to pyruvate while regenerating ATP by pyruvate kinase. This reaction is followed by conversion of pyruvate to lactate by lactate dehydrogenase which is coupled to the oxidation of NADH to NAD^+^. Thus, the ATPase rate can be calculated from the decrease in NADH absorption at 340 nm over time. Assays were performed in a Jasco V-650 spectrophotometer for 30 min in 150 μL Min buffer using the following concentrations of components: 4 μM MinD, 4 μM MinE, 0.2 mg/mL small unilamellar vesicles of *E*. *coli* polar lipids, 0.5 mM NADH (Sigma–Aldrich, St. Louis, MO, USA), 2 mM PEP (Sigma–Aldrich, St. Louis, MO, USA), 24 U/mL pyruvate kinase and 35 U/mL lactate dehydrogenase (enzyme mix from Sigma–Aldrich, St. Louis, MO, USA) as well as 1 mM ATP.

## Supporting information

S1 Fig Truncation or mutation of MinE’s membrane targeting sequence disrupts interaction of MinE with lipid membranes *in vitro*. **(A)** As MinE membrane interaction requires exposure of its MTS through sensing membrane-bound MinD, the effect of MTS mutations was tested in the background of the I24N mutation. The I24N mutation mimics and bypasses MinE’s interaction with membrane-bound MinD, exposing MinE’s MTS and thus allowing the detection of MinE’s interaction with the membrane independent of its interaction with MinD [31]. Structures correspond to closed *N*. *gonorrhoeae* MinE (PDB 2KXO) and open *E*. *coli* MinE (PDB 3R9J) with the I24N mutation highlighted in yellow. **(B)** Representative SDS-PAGE fractions from co-sedimentation experiments of MinE I24N mutants with small unilamellar vesicles. SN: supernatant. P: pellet. **(C)** Percentage of pelleted protein for MinE I24N in the presence or absence of additional MTS mutations. Error bars represent standard deviation from three independent experiments.
(TIF)

S2 Fig Bidirectional rotations emerging with MinE L3E appear like an oscillation along the minor axis. The kymograph along the compartment width and time-averaged fluorescence intensity, measured in the rectangular area highlighted below, are plotted for the same compartment exhibiting bidirectional rotations shown in Fig 3.
(TIF)

S3 Fig Unusual dynamics observed with MinE ∆(2–12). All images at 1 μM MinD with 20% eGFP-MinD and 1 μM MinE. Time-averaged protein distributions were measured as in Fig 3. Scale Bar: 5 μm.
(TIF)

S4 Fig Unusual dynamics observed with MinE F6E. All images at 1 μM MinD with 20% eGFP-MinD and 1 μM MinE. Time-averaged protein distributions were measured as in Fig 3. Scale Bar: 5 μm.
(TIF)

S5 Fig Relative fractions of observed modes for MinE Δ(2–12), L3E and F6E. Bi- and unidirectional rotations were classified together, as they were sometimes difficult to distinguish. Chaotic dynamics, which occasionally occurred but could not be clearly assigned, were not taken into account.
(TIF)

S1 Table Primers used to generate mutations in MinE. (PDF)

S2 Table Absolute numbers of different dynamic modes observed for WT MinE and MinE Δ(2–12), L3E and F6E in PDMS microcompartments. Modes were counted in three independent experiments imaging multiple compartments respectively (N ≥ 55 compartments). If mode switching occurred within the same compartment, both modes were counted.
(PDF)

S1 Movie Confocal time-lapse movie of spiral waves emerging with MinE Δ(2–12) on flat membranes. Protein concentrations: 1 μM MinD with 20% eGFP-MinD, 1 μM MinE Δ(2–12). The movie follows the dynamics for around 3 min 30 s.
(MOV)

S2 Movie Confocal time-lapse movie of pole-to-pole oscillations with WT MinE in a cell-shaped compartment. Protein concentrations: 1 μM MinD with 20% eGFP-MinD, 1 μM WT MinE. The movie follows the dynamics for 4 min. Scale Bar: 5 μm.
(MOV)

S3 Movie Confocal time-lapse movie of bidirectional rotations with MinE L3E in a cell-shaped compartment. Protein concentrations: 1 μM MinD with 20% eGFP-MinD, 1 μM MinE L3E. The movie follows the dynamics for 4 min. Scale Bar: 5 μm.
(MOV)

S4 Movie Confocal time-lapse movie of unidirectional rotations with MinE L3E in a cell-shaped compartment. Protein concentrations: 1 μM MinD with 20% eGFP-MinD, 1 μM MinE L3E. The movie follows the dynamics for 4 min. Scale Bar: 5 μm.
(MOV)

S5 Movie Confocal time-lapse movie of traveling wave dynamics with MinE L3E in a cell-shaped compartment. Protein concentrations: 1 μM MinD with 20% eGFP-MinD, 1 μM MinE L3E. The movie follows the dynamics for 4 min. Scale Bar: 5 μm.
(MOV)

S6 Movie Confocal time-lapse movie of pole-to-pole dynamics with MinE L3E in a cell-shaped compartment. Protein concentrations: 1 μM MinD with 20% eGFP-MinD, 1 μM MinE L3E. The movie follows the dynamics for 4 min. Scale Bar: 5 μm.
(MOV)
