# Supplementary figures and images for: Large-scale modulation of reconstituted Min protein patterns and gradients by defined mutations in MinE’s membrane targeting sequence
Source: PLoS One. 2017 Jun 16;12(6):e0179582. doi: 10.1371/journal.pone.0179582 (PMC5473585; doi:10.1371/journal.pone.0179582)

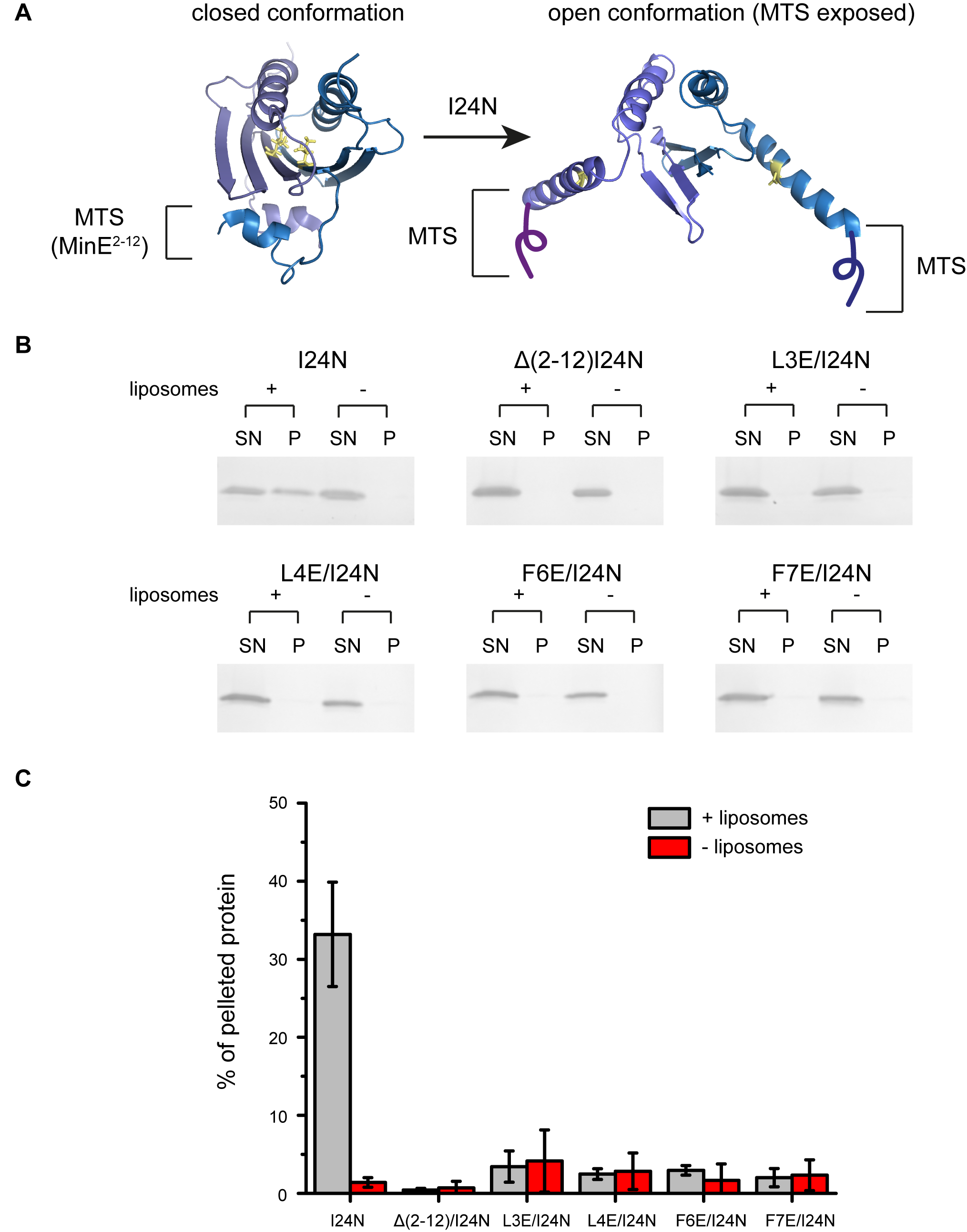

Supplement: S1 Fig — (A) As MinE membrane interaction requires exposure of its MTS through sensing membrane-bound MinD, the effect of MTS mutations was tested in the background of the I24N mutation. The I24N mutation mimics and bypasses MinE’s interaction with membrane-bound MinD, exposing MinE’s MTS and thus allowing the detection of MinE’s interaction with the membrane independent of its interaction with MinD [31]. Structures correspond to closed N. gonorrhoeae MinE (PDB 2KXO) and open E. coli MinE (PDB 3R9J) with the I24N mutation highlighted in yellow. (B) Representative SDS-PAGE fractions from co-sedimentation experiments of MinE I24N mutants with small unilamellar vesicles. SN: supernatant. P: pellet. (C) Percentage of pelleted protein for MinE I24N in the presence or absence of additional MTS mutations. Error bars represent standard deviation from three independent experiments. (TIF) [file pone.0179582.s001.tif]

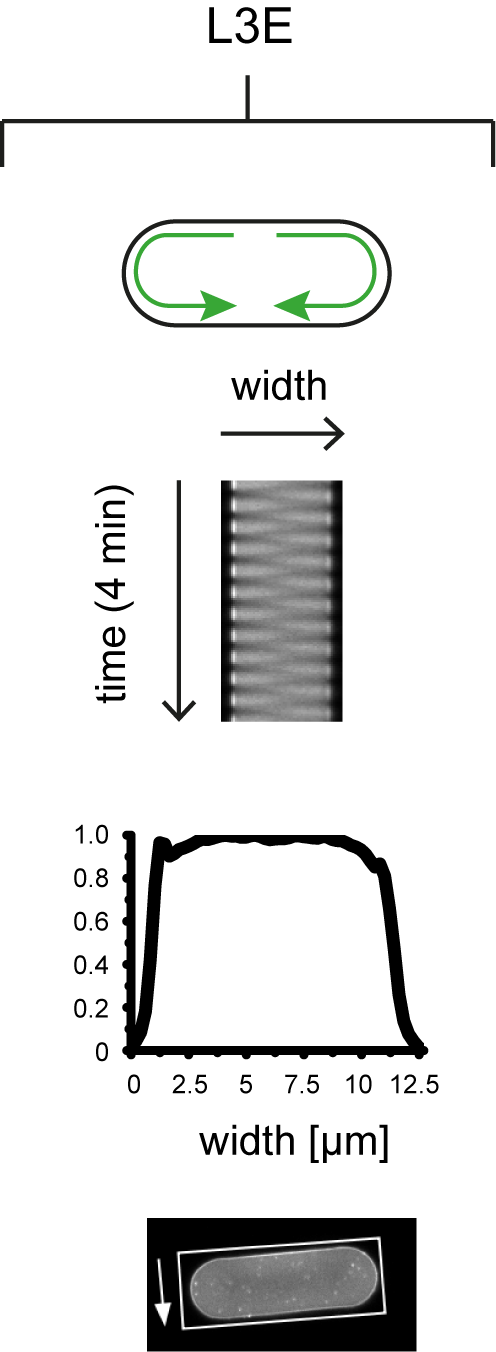

Supplement: S2 Fig — The kymograph along the compartment width and time-averaged fluorescence intensity, measured in the rectangular area highlighted below, are plotted for the same compartment exhibiting bidirectional rotations shown in Fig 3. (TIF) [file pone.0179582.s002.tif]

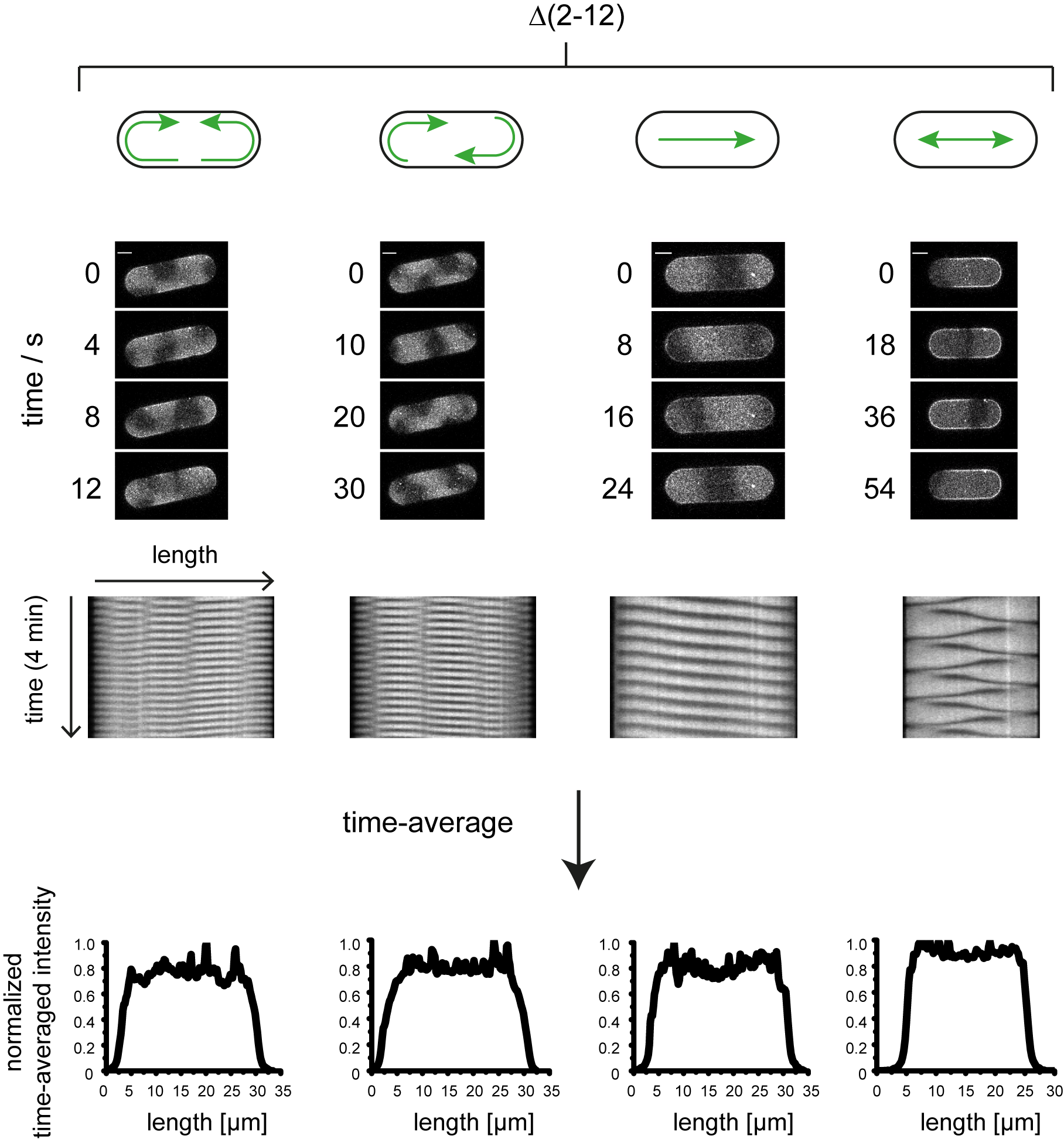

Supplement: S3 Fig — All images at 1 μM MinD with 20% eGFP-MinD and 1 μM MinE. Time-averaged protein distributions were measured as in Fig 3. Scale Bar: 5 μm. (TIF) [file pone.0179582.s003.tif]

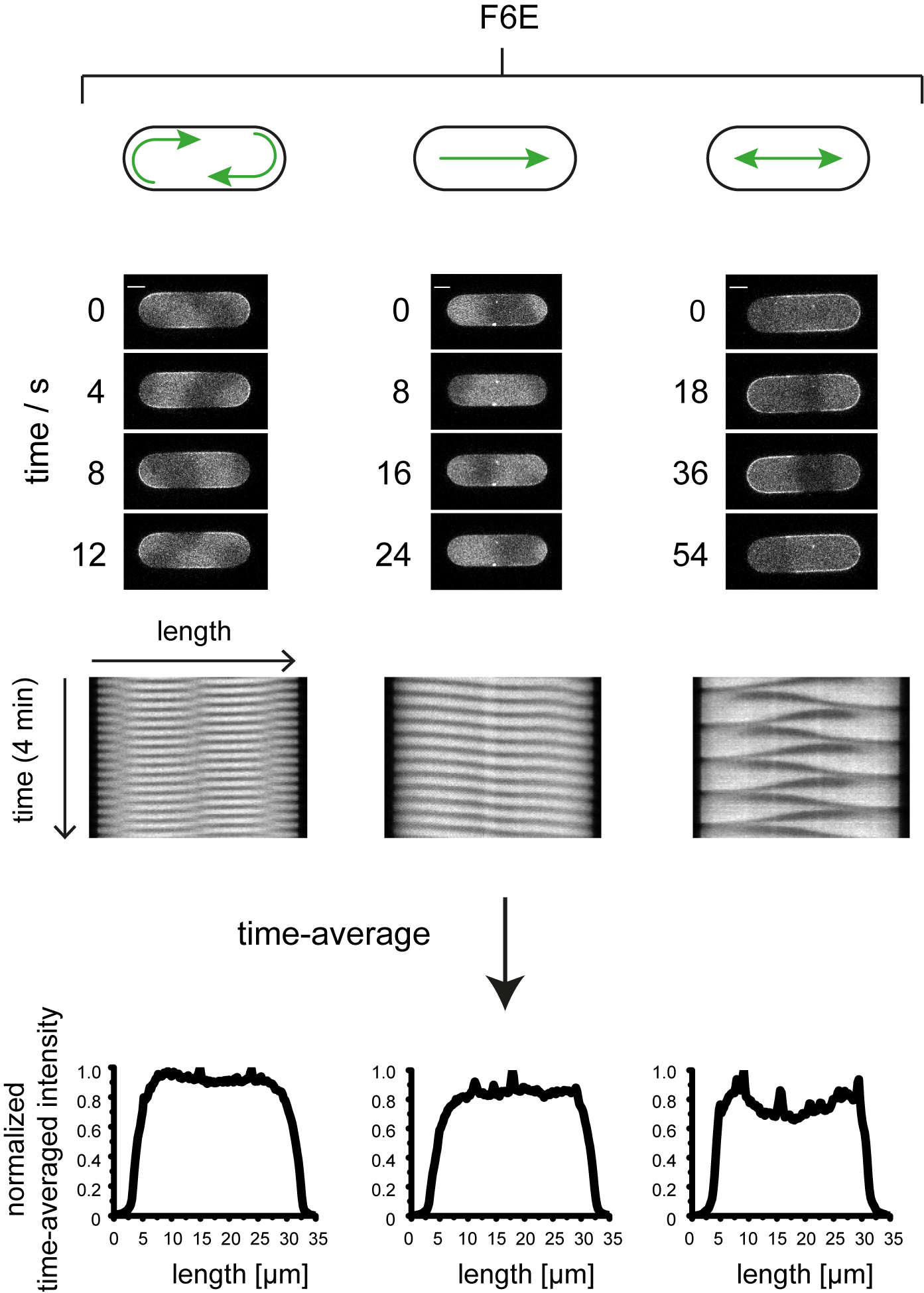

Supplement: S4 Fig — All images at 1 μM MinD with 20% eGFP-MinD and 1 μM MinE. Time-averaged protein distributions were measured as in Fig 3. Scale Bar: 5 μm. (TIF) [file pone.0179582.s004.tif]

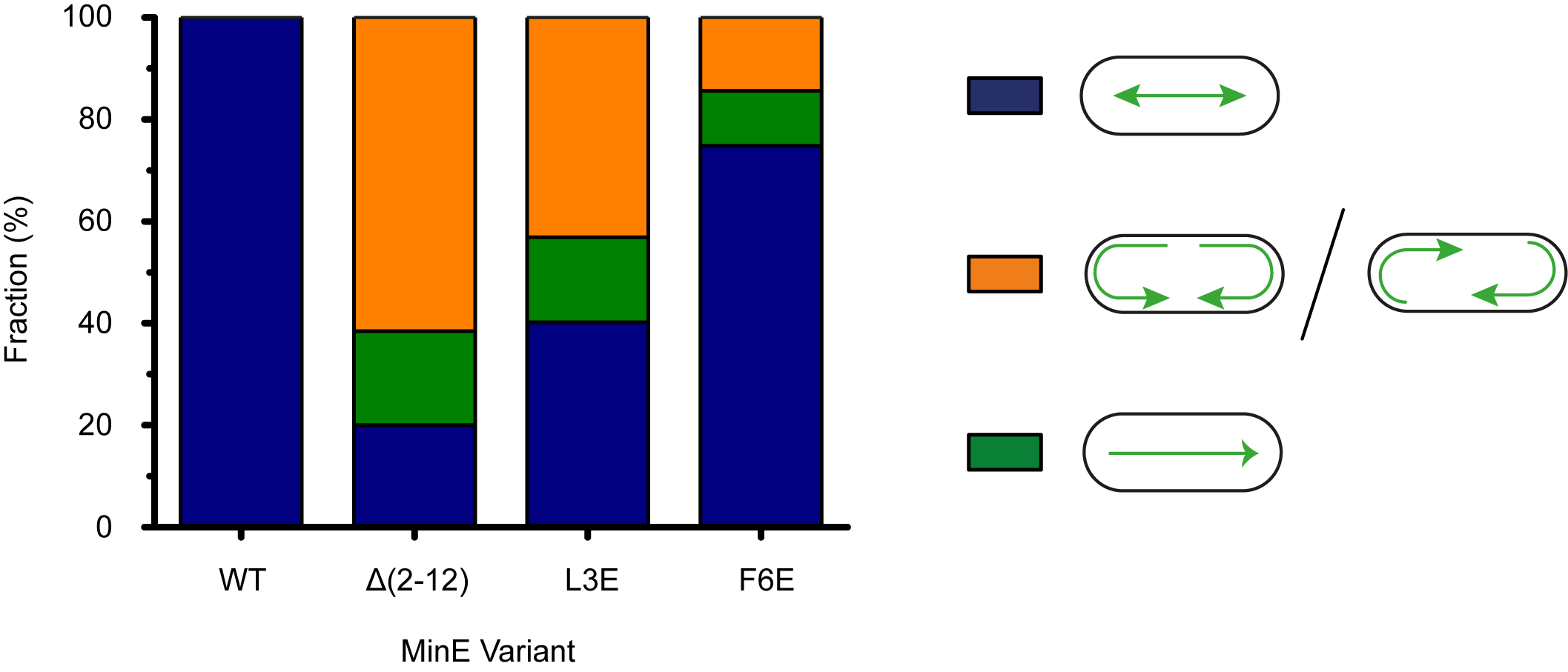

Supplement: S5 Fig — Bi- and unidirectional rotations were classified together, as they were sometimes difficult to distinguish. Chaotic dynamics, which occasionally occurred but could not be clearly assigned, were not taken into account. (TIF) [file pone.0179582.s005.tif]
