# Supplementary material for: Large-scale modulation of reconstituted Min protein patterns and gradients by defined mutations in MinE’s membrane targeting sequence
Source: PLoS One. 2017 Jun 16;12(6):e0179582. doi: 10.1371/journal.pone.0179582 (PMC5473585; doi:10.1371/journal.pone.0179582)
Supplement: S1 Table — (PDF) [file pone.0179582.s006.pdf]

S1 Table. Primers used to generate mutations in MinE

**Primer name**	**Sequence (5'-3')**
MinE_L3E_fwd	GGATCCGAATTCGCAGAACTCGATTTCTTTCT
MinE_L3E_rev	AGAAAGAAATCGAGTTCTGCGAATTCGGATCC
MinE_L4E_fwd	TCCGAATTCGCATTAGAAGATTTCTTTCTCTCG
MinE_L4E_rev	CGAGAGAAAGAAATCTTCTAATGCGAATTCGGA
MinE_F6E_fwd	TTCGCATTACTCGATGAATTTCTCTCGCGGAAG
MinE_F6E_rev	CTTCCGCGAGAGAAATTCATCGAGTAATGCGAA
MinE_F7E_fwd	GCATTACTCGATTTCGAACTCTCGCGGAAGAAA
MinE_F7E_rev	TTTCTTCCGCGAGAGTTCGAAATCGAGTAATGC
MinE_Δ(2-12)_fwd_1	CGCGGATCCGAATTCAACACAGCCAACATTGCAAAAGAA
MinE_Δ(2-12)_rev_1	AATGTTGGCTGTGTTGAATTCGGATCCGCGACCCATTTG
MinE_Δ(2-12)_fwd_2	CCGCCTTTGAGTGAGCTGATACCGCTCGCCGCAGCCGAA
MinE_Δ(2-12)_rev_2	CTCACTCAAAGGCGGTAATACGGTTATCCACAGAATCAG
MinE_I24N_fwd	AAGAACGGCTGCAGAACATTGTTGCTGAACGC
MinE_I24N_rev	GCGTTCAGCAACAATGTTCTGCAGCCGTTCTT
