# Supplementary material for: Large-scale modulation of reconstituted Min protein patterns and gradients by defined mutations in MinE’s membrane targeting sequence
Source: PLoS One. 2017 Jun 16;12(6):e0179582. doi: 10.1371/journal.pone.0179582 (PMC5473585; doi:10.1371/journal.pone.0179582)
Supplement: S2 Table — Modes were counted in three independent experiments imaging multiple compartments respectively (N ≥ 55 compartments). If mode switching occurred within the same compartment, both modes were counted. (PDF) [file pone.0179582.s007.pdf]

**S2 Table. Absolute numbers of different dynamic modes observed for WT MinE and MinE Δ(2-12), L3E and F6E in PDMS microcompartments.** Modes were counted in three independent experiments imaging multiple compartments respectively (N ≥ 55 compartments). If mode switching occurred within the same compartment, both modes were counted.

| **MinE Variant** | **Pole-to-pole oscillations** | **Traveling waves** | **Rotations** |
|---|---|---|---|
| **WT** | 81 | 0 | 0 |
| **Δ(2-12)** | 13 | 12 | 40 |
| **L3E** | 41 | 17 | 44 |
| **F6E** | 104 | 15 | 20 |
